# Treatment Responsiveness in CIDP Patients with Diabetes Is Associated with Higher Degrees of Demyelination

**DOI:** 10.1371/journal.pone.0139674

**Published:** 2015-10-13

**Authors:** Alon Abraham, Majed Alabdali, Mohammad Qrimli, Hana Albulaihe, Ari Breiner, Carolina Barnett, Hans D. Katzberg, Leif E. Lovblom, Bruce A. Perkins, Vera Bril

**Affiliations:** 1 Ellen and Martin Prosserman Centre for Neuromuscular Diseases, Division of Neurology, Department of Medicine, University Health Network, University of Toronto, Toronto, Canada; 2 Department of Neurology, King Fahad Hospital of the University, University Of Dammam, Dammam, Saudi Arabia; 3 Department of Neurology, King Khalid University Hospital, King Saud University, Riyadh, Saudi Arabia; 4 Division of Endocrinology and Metabolism, Department of Medicine, Mount Sinai, Hospital and Lunenfeld Tanenbaum Research Institute, University of Toronto, Toronto, Canada; University of Würzburg, GERMANY

## Abstract

**Introduction:**

Chronic inflammatory demyelinating polyradiculoneuropathy (CIDP) is one of several chronic treatable acquired demyelinating neuropathies.

**Objectives:**

To explore the association between the degree of demyelination in CIDP, and treatment responsiveness.

**Methods:**

A retrospective chart review of CIDP subjects assessed between 1997 and 2013 was performed to compare treatment responsiveness using different sets of criteria.

**Results:**

99 CIDP patients were included, 34 with diabetes mellitus (DM). Treatment responsiveness was higher in CIDP-DM fulfilling 1 or more EFNS/PNS criteria, (63% vs. 31%, p = 0.03), and in CIDP+DM fulfilling 2 or more criteria (89% vs. 36%, p = 0.01). Nonetheless, treatment responsiveness in CIDP+DM had the highest odds ratio (3.73, p = 0.01). Similar results were also shown in simplified uniform study criteria, with 10% cut off values for CIDP-DM, compared to 30% for CIDP+DM.

**Conclusion:**

In CIDP+DM, higher degrees of demyelination are associated with treatment responsiveness, implying the need to adjust current criteria in these patients.

## Introduction

Chronic inflammatory demyelinating polyradiculoneuropathy (CIDP) is one of several chronic treatable acquired demyelinating neuropathies. There are currently 15 sets of criteria using a variable combination of clinical, electrophysiological, laboratory, and biopsy features to identify CIDP [1]. Although those criteria might be appropriate in the research setting, such rigorous electrophysiological criteria lack sensitivity for the diagnosis, and may miss clinical cases of CIDP[2]. As a result, there are currently no widely accepted practical clinical criteria for diagnosing CIDP[3].

The American Academy of Neurology (AAN) research criteria are highly specific, but lack sensitivity[4], so many patients who are diagnosed clinically with CIDP by clinicians do not meet these criteria. The European Federation of Neurological Societies/Peripheral Nerve Society (EFNS/PNS) consensus guideline was designed to offer diagnostic criteria with greater sensitivity in clinical practice, in order to avoid missing patients with this treatable disease[5], and are the most frequently used criteria[6]. Generally speaking, the EFNS/PNS criteria include distal latency prolongation 50% above the upper normal limit, reduction of motor conduction velocity 30% below the lower normal limit, prolongation of F-wave latency 30% above the upper normal limit, conduction block, temporal dispersion, and absence of F-waves in at least two motor nerves[7]. The most widely used treatments for CIDP consist of intravenous immune globulin, plasma exchange, and corticosteroids, with improvement in 60 to 80 percent of patients[8]. However, response to treatment is difficult to predict. Prediction of treatment outcome has been related to the pattern of weakness[9], disease duration[10], the presence of monoclonal gammopathy[11], distribution patterns of conduction abnormalities[7,12], and the selection of electrodiagnostic criteria[13]. However, whether fulfilling CIDP electrophysiological criteria can predict treatment response rates is controversial[13,14].

Our aim in this study was to further explore the association between treatment response rates and the degree of demyelination in CIDP patients with and without diabetes, as measured by number of criteria fulfilled, using different sets of criteria for CIDP diagnosis, including EFNS/PNS and AAN criteria, as well as simplified uniform study criteria with different cut off values for electrophysiological parameters of 10%, 20%, and 30%.

## Materials and Methods

### Subjects

CIDP subjects attending the Neuromuscular clinic for management of their immune-mediated polyneuropathy between 1997 and 2013 were evaluated for this study. In this retrospective review we extracted demographic data, the clinical history, physical examination, laboratory test results, and electrophysiological data from previously coded charts of CIDP patients. The Research Ethics Board of the University Health Network approved the current study protocol, based on chart review and collection of de-identified data.

A cohort of CIDP+DM treated patients (n = 34), and age and gender matched CIDP-DM treated patients (n = 65) from a previous study was used [15]. The diagnosis of DM was confirmed according to the American Association of Diabetes criteria based on one of four abnormalities: haemoglobin A1c, fasting plasma glucose, random elevated glucose with symptoms, or abnormal 2 hour oral glucose tolerance test[16]. CIDP was diagnosed according to the Koski criteria that require either clinical or eletrophysiological features suggestive of CIDP based on the clinical presentation, as judged by a neuromuscular expert (VB), and the presence of demyelination on NCS[17]. This study excluded patients with proximal diabetic radiculoplexus neuropathies based on the typical clinical presentation; i.e.: patients presenting with asymmetrical, acute proximal leg pain followed by weakness, as the pain resolved. Although, atypical painless diabetic radiculoplexus neuropathies, with more symmetrical lower limb, and some with upper limb involvement, due to ischemic injury and microvasculitis, have also been described, these patients have axonal neuropathies and were excluded on clinical and electrophysiological criteria[18]. The duration of polyneuropathy was taken as the time from when the patient first developed symptoms to their evaluation in the Neuromuscular clinic.

CIDP patients, with or without DM, were also classified as responders (n = 53) or non- responders (n = 46) based on clinical response to treatment assessed by a combination of patient and physician evaluations in the 99 patients who were treated with immunotherapies. Responders were those who stabilized after declining progressively, or improved after treatment, and non-responders either worsened or did not change after treatment based on their clinical evaluation.

Subjects were evaluated for neuropathy by neurological examination, the 19-point Toronto Clinical Neuropathy Score (TCNS), vibration perception threshold (VPT), and median, ulnar, peroneal, tibial, and sural NCS[17,19]. NCS were performed using the Sierra Wave instrument (Cadwell Laboratories Inc., Kennewick, WA, USA). Age- and height-adjusted NCS reference values were used, according to the standards of the Toronto General Hospital (University Health Network) electromyography laboratory. Limb temperature was measured prior to nerve conduction studies, and if required, warming was performed to ensure a surface temperature of ≥32.0°C in the upper limbs and ≥31.0°C in the lower limbs.

Median, ulnar, peroneal, tibial and sural NCS were performed using surface stimulating and recording techniques according to the standards of the Canadian Society of Clinical Neurophysiology and the American Association of Neuromuscular and Electrodiagnostic Medicine[20]. The electromyography Instrument calculated latencies, amplitudes and conduction velocities automatically. Median, ulnar, peroneal and tibial nerve motor amplitudes were measured as baseline to peak for the CMAP, and from baseline to negative peak for the sural sensory nerve action potential (SNAP) amplitude, or from the positive peak (if present) to the negative peak. The sural nerve latency was measured at the onset of the initial deflection from baseline. The F wave latency was determined as the minimum reproducible latency obtained after 10 supramaximal stimuli were applied to the median and ulnar nerves at the wrist, and tibial and peroneal nerves at the ankle.

For the purpose of this study, we compared response rates in CIDP patients with and without DM, fulfilling different criteria to diagnose CIDP from 2 different sets of previously published criteria, including the AAN[4], and the EFNS/PNS criteria[7], as well as from 3 additional sets of study criteria. In these additional sets, which were simplified and uniform for different demyelinative features, each criteria set was defined by a similar degree of demyelinating changes, measured by percentages, with different cut off values, of at least 10%, 20%, or 30%, for prolonged distal or F wave latencies, or slowed conduction velocities. For all of the simplified and uniform criteria conduction block was defined as the presence of a 50% amplitude reduction from distal to proximal sites, in at least 2 nerves.

### Statistical analysis

Statistical analysis was performed using SAS version 9.2 for Windows (SAS Institute, Cary, North Carolina). Clinical and electrophysiological characteristics were expressed as means ± standard deviations (SD) for continuous data, or as frequency and percent for ordinal data. Comparisons between treatment responders and non-responders were made using the student’s t-test, the Wilcoxon rank-sum test, or the χ2-test, depending on the type and distribution of the variable. Normality was tested using the Shapiro-Wilk test. The number of criteria met for each of the EFNS/PNS, AAN, and study criteria definitions of CIDP were treated as ordinal variables, and univariable logistic regression models were run using responder status as the dependent variable, and “number of criteria met” as the independent variables. We report the regression coefficients, p-values, and odds ratios for each independent variable. Additionally, receiver operating characteristic (ROC) curves and the corresponding area-under-the-curve (AUC) using Responder Status as the gold-standard measure were generated for each independent variable. The analysis was completed for the entire cohort, as well as for the diabetes and non-diabetes subgroups. Significance was set at α-level of 0.05.

## Results

We examined 99 CIDP subjects, 34 with DM, and 65 without diabetes (S1 File). Their demographic, clinical and electrophysiological characteristics are described elsewhere[21]. While 53 CIDP patients responded to treatment, 46 did not. CIDP patients responding to treatment fulfilled more EFNS/PNS criteria (1.6±1.2 vs. 0.9±1, p = 0.001), 10% cut off study criteria (1.9± 1 vs. 1.4±1, p = 0.02), and 30% cut off study criteria (1.1±1 vs. 0.6±0.9, p = 0.01), with only a trend toward fulfilling more AAN criteria (1.4±1.3 vs. 0.9±1.1, p = 0.05). CIDP-DM patients fulfilled more EFNS/PNS criteria (1.6±1.1 vs. 1±1.1, p = 0.03), AAN criteria (1.5±1.4 vs. 0.8±1.2, p = 0.04), and 10% study criteria (1.9± 1 vs. 1.3±1.1, p = 0.02), while CIDP+DM patients fulfilled more EFNS/PNS criteria (1.4±0.9 vs. 0.6±0.6, p = 0.01), and 30% study criteria (1.2± 1 vs. 0.5±0.6, p = 0.04) (Table 1).

**Table 1:** Comparing the number of EFNS, AAN, and study criteria met between treatment responders and non-responders.

	Treatment response		
Criteria	No	Yes	p-value
***Whole Cohort***			
N	46	53	
EFNS	0.9±1.0	1.6±1.2	**0.001**
AAN	0.9±1.1	1.4±1.3	0.05
10%*	1.4±1.0	1.9±1.0	**0.02**
20%*	0.9±1.0	1.3±1.0	0.08
30%**	0.6±0.9	1.1±1.0	**0.01**
***CIDP-DM***			
N	29	36	
EFNS	1.0±1.1	1.6±1.2	**0.03**
AAN	0.8±1.2	1.5±1.4	**0.04**
10%*	1.3±1.1	1.9±1.0	**0.02**
20%*	0.9±1.0	1.3±1.1	0.08
30%**	0.6±1.0	1.0±1.1	0.08
***CIDP+DM***			
N	17	17	
EFNS	0.6±0.6	1.4±0.9	**0.01**
AAN	0.9±1.0	1.1±1.2	0.74
10%*	1.6±0.9	1.8±1.1	0.42
20%*	1.1±0.8	1.2±1.0	0.54
30%**	0.5±0.6	1.2±1.0	**0.04**

Mean and standard deviation are indicated.

CIDP: Chronic inflammatory demyelinating polyradiculoneuropathy; DM: Diabetes mellitus

EFNS criteria include one of four demyelinating features: reduction in conduction velocity, distal latency or F wave prolongation, or conduction blocks in 2 nerves. AAN criteria include three of four demyelinating features: reduction in conduction velocity, distal latency or F wave prolongation, or conduction blocks in 2 nerves.

* Study parameters, including 10% reduction in conduction velocity, distal latency or F wave prolongation, and conduction blocks, from any of the nerves tested.

** Study parameters, including 30% reduction in conduction velocity, distal latency or F wave prolongation, and conduction blocks, from any of the nerves tested.

Fulfilling 2 or more criteria, was associated with statistically significant higher treatment response rates in the whole cohort for all studied criteria, including EFNS/PNS, 10%, and 30% cut off study criteria, except for the 20% cut off study criteria. In CIDP-DM patients the higher treatment response rates were observed only for those fulfilling 1 or more criteria (63% vs. 31%, p = 0.03), whereas this was demonstrated in CIDP+DM patients fulfilling 2 or more criteria (89% vs. 36%, p = 0.01). Similarly, higher treatment response rates in CIDP-DM patients were associated only for those fulfilling 2 or more 10% cut off study criteria (67% vs. 35%, p = 0.01), whereas this association was demonstrated in CIDP+DM patients fulfilling 2 or more 30% cut off study criteria (89% vs. 36%, p = 0.01)(Table 2).

**Table 2:** Response-to-therapy rates based on different numbers of EFNS, AAN, and study criteria met.

Criteria		Treatment Responders % (n)		
		Whole Cohort	CIDP-DM	CIDP+DM
EFNS	<1	30% (8/27)	31% (5/16)	27% (3/11)
	≥1	63% (45/72)	63% (31/49)	61% (14/23)
	p value	**0.004**	**0.03**	0.07
	<2	43% (28/65)	48% (19/40)	36% (9/25)
	≥2	74% (25/34)	68% (17/25)	89% (8/9)
	p value	**0.004**	0.11	**0.01**
AAN	<1	47% (20/43)	43% (12/28)	53% (8/15)
	≥1	59% (33/56)	65% (24/37)	47% (9/19)
	p value	0.22	0.08	0.73
	<2	44% (28/64)	45% (19/42)	41% (9/22)
	≥2	71% (25/35)	74% (17/23)	67% (8/12)
	p value	**0.01**	**0.03**	0.15
10%*	<1	39% (7/18)	33% (4/12)	50% (3/6)
	≥1	57% (46/81)	60% (32/53)	50% (14/28)
	p value	0.17	0.09	1.00
	<2	38% (13/34)	35% (8/23)	45% (5/11)
	≥2	62% (40/65)	67% (28/42)	52% (12/23)
	p value	**0.03**	**0.01**	0.71
30%**	<1	42% (21/50)	44% (15/34)	38% (6/16)
	≥1	65% (32/49)	68% (21/31)	61% (11/18)
	p value	**0.02**	0.06	0.17
	<2	46% (32/69)	52% (23/44)	36% (9/25)
	≥2	70% (21/30)	62% (13/21)	89% (8/9)
	p value	**0.03**	0.47	**0.01**

CIDP: Chronic inflammatory demyelinating polyradiculoneuropathy; DM: Diabetes mellitus; AAN: American Academy of Neurology; EFNS: European Federation of Neurological Societies.

* Study parameters, including 10% reduction in conduction velocity, distal latency or F wave prolongation, and conduction blocks, from any of the nerves tested.

** Study parameters, including 30% reduction in conduction velocity, distal latency or F wave prolongation, and conduction blocks, from any of the nerves tested.

CIDP patients fulfilling any of the studied criteria (excluding the 20% cut off study criteria) had higher treatment response rates, using EFNS/PNS criteria (Odds ratio (OR) = 1.83, p = 0.003), AAN criteria (OR = 1.42, p = 0.04), 10% cut off study criteria (OR = 1.60, p = 0.02), and 30% cut off study criteria (OR = 1.77, p = 0.01). Although the highest OR was found in CIDP+DM patients fulfilling EFNS/PNS criteria (3.73, p = 0.01), with treatment response rates increasing consistently from 27% to 100% in concordance with the number of EFNS criteria fulfilled, in CIDP-DM patients only a trend was observed with a lower OR (OR = 1.53, p = 0.05). Higher treatment response rates in patients fulfilling different study criteria were different between subgroups. In CIDP-DM patients, only those fulfilling 10% cut off study criteria (OR = 1.8, p = 0.02), and in CIDP+DM patients, only those fulfilling 30% cut off study criteria (OR = 2.73, p = 0.03)(Table 3), showed higher treatment response ratios. The OR was not statistically significant in all groups fulfilling 20% study criteria (Table 1).

**Table 3:** Response-to-therapy rates based on numbers of EFNS, AAN, and study criteria met.

Criteria		Treatment Responders % (n)		
		Whole Cohort	CIDP-DM	CIDP+DM
EFNS	0	30% (8/27)	31% (5/16)	27% (3/11)
	1	53% (20/38)	58% (14/24)	43% (6/14)
	2	72% (13/18)	67% (8/12)	83% (5/6)
	3	78% (7/9)	67% (4/6)	100% (3/3)
	4	71% (5/7)	71% (5/7)	-
	OR (CI)	1.83 (1.22,2.74)	1.53 (0.99,2.36)	3.73 (1.32,10.60)
	p	**0.003**	0.05	**0.01**
AAN	0	47% (20/43)	43% (12/28)	53% (8/15)
	1	38% (8/21)	50% (7/14)	14% (1/7)
	2	76% (13/17)	78% (7/9)	75% (6/8)
	3	62% (8/13)	67% (6/9)	50% (2/4)
	4	80% (4/5)	80% (4/5)	-
	OR (CI)	1.42 (1.02,2.00)	1.54 (1.02,2.31)	1.17 (0.62,2.20)
	p	**0.04**	**0.04**	0.63
10%*	0	39% (7/18)	33% (4/12)	50% (3/6)
	1	35% (7/20)	33% (5/15)	40% (2/5)
	2	63% (24/38)	77% (17/22)	44% (7/16)
	3	64% (14/22)	60% (9/15)	71% (5/7)
	4	100% (1/1)	100% (1/1)	-
	OR (CI)	1.60 (1.07,2.39)	1.80 (1.09,2.96)	1.28 (0.64,2.55)
	p	**0.02**	**0.02**	0.49
30%**	0	42% (21/50)	44% (15/34)	38% (6/16)
	1	23% (12/22)	69% (9/13)	33% (3/9)
	2	75% (15/20)	67% (8/12)	88% (7/8)
	3	71% (5/7)	67% (4/6)	100% (1/1)
	OR (CI)	1.77 (1.13,2.75)	1.50 (0.90,2.50)	2.73 (1.11,6.73)
	p	**0.01**	0.12	**0.03**

CIDP: Chronic inflammatory demyelinating polyradiculoneuropathy; DM: Diabetes mellitus; AAN: American Academy of Neurology; EFNS: European Federation of Neurological Societies.

OR: Odds ratio, calculated by llogistic regression, with treatment responder (yes or no) as the dependent variable.

* Study parameters, including 10% reduction in conduction velocity, distal latency or F wave prolongation, and conduction blocks, from any of the nerves tested.

** Study parameters, including 30% reduction in conduction velocity, distal latency or F wave prolongation, and conduction blocks, from any of the nerves tested.

**Fig 1:**
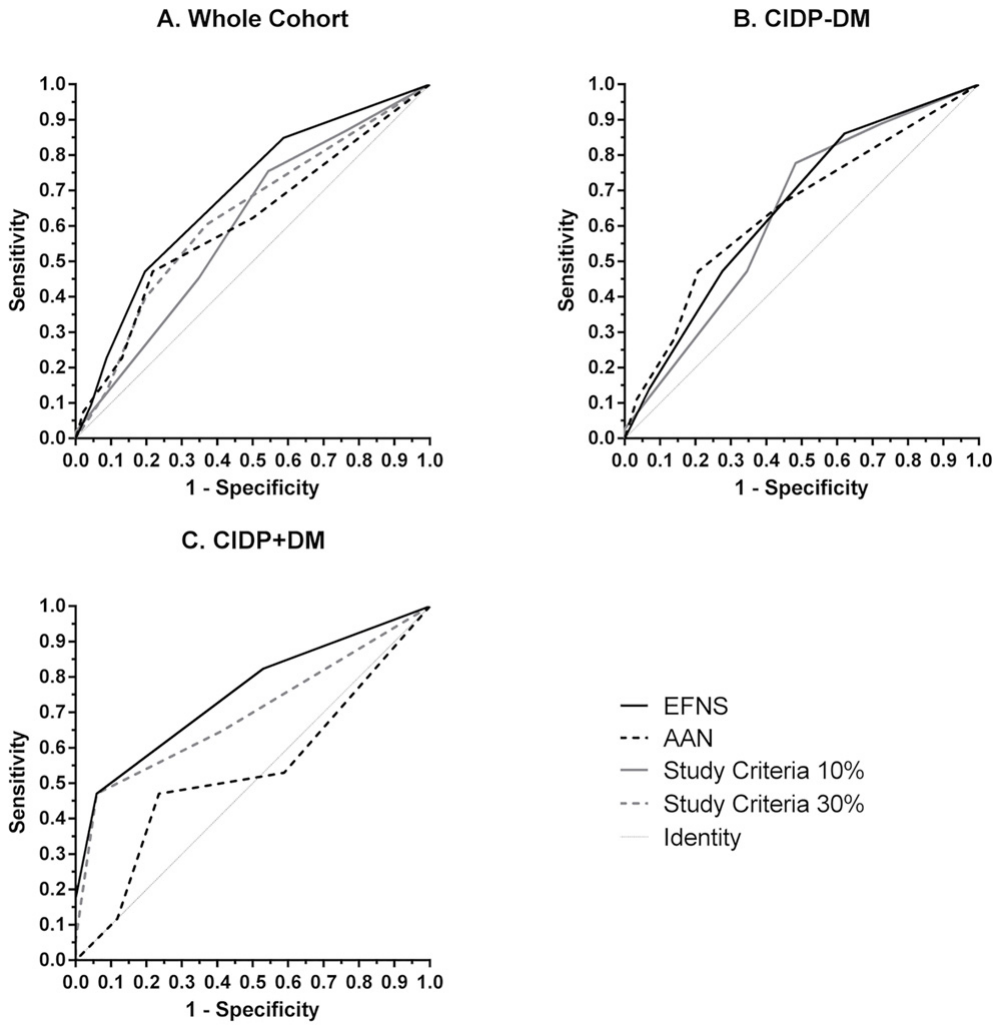
ROC curves for EFNS, AAN, and study criteria. (A) Whole cohort. (B) CIDP-DM patients. (C) CIDP+DM patients.

Areas under receiver operator characteristics (ROC) curves for EFNS/PNS, AAN, and study criteria, ranged from 0.53 to 0.73. Those that were statistically significant included EFNS criteria, generally the highest (0.68 (p = 0.002) whole cohort, 0.65 (p = 0.04) CIDP-DM patients subgroup, and 0.73 (p = 0.02) CIDP+DM patients), 10% and 30% cut off study criteria for the whole cohort 0.63, and 0.64 (p = 0.02), 10% cut off study criteria for CIDP-DM patients, 0.66 (p = 0.02), and a trend at the 30% cut off study criteria in CIDP+DM patients subgroup 0.7 (p = 0.05). All areas under ROC curves using AAN criteria were not statistically significant (Fig 1).

## Discussion

Although CIDP+DM and CIDP-DM patients have similar treatment response rates[15,22–24], the degree of improvement might be less favourable in CIDP+DM patients, probably due to additive effects of superimposed diabetic distal symmetric sensorimotor polyneuropathy (DSP)[22]. Whether fulfilling various electrophysiological criteria for CIDP can predict treatment response rates in these patients, is controversial[13,14]; however, we have shown in a previous study that fulfilling EFNS/PNS, but not AAN criteria, is associated with higher treatment response rates in CIDP patients. Similar results were shown also in CIDP-DM patients, but not in CIDP+DM patients[21]. In the current study we further explored the association between fulfilling different numbers of criteria and treatment response rates.

The major finding of the current study, is that fulfilling a higher number of electrophysiological criteria in CIDP patients, with and without DM, is associated with higher treatment response rates. An association between treatment responsiveness and demyelination has been reported previously, and the presence of axonal features predicted lack of response[25]. However, in contrast to the association between relatively mild degrees of demyelination and higher treatment response rates in CIDP patients without DM, only higher degrees of demyelination are associated with higher treatment response rates in those patients with DM. The implication is that a single EFNS criterion, which is sufficient to consider CIDP and offer patients treatment, predicts higher treatment response rates in CIDP patients without DM, whereas 2 criteria need to be present in order to predict higher treatment response rates in CIDP patients with DM.

A possible explanation for the finding that only relatively high degrees of demyelination are associated with higher treatment response rates in CIDP+DM patients, might be related to underlying DSP. The coexistence of DSP was indicated by lower sural sensory nerve action potential (SNAP) amplitudes in CIDP+DM patients compared to CIDP-DM patients (2.3 μV vs. 6.8 μV, p<0.0001)[21]. The presence of DSP might lead to misclassification, as DSP may cause conduction slowing attributable to loss of the fastest conducting large myelinated fibers, although this is usually considered to be mild, and seldom fulfils electrophysiological criteria for CIDP[26]. Nonetheless, although the pathophysiology of DSP is mainly axonal degeneration with progressive loss of nerve fibers, resulting in reduction of the amplitudes of the sensory and motor responses[27–30], demyelinating features are described in DSP, with conduction velocity slowing that cannot be explained by axon loss alone[31,32], and significant amplitude independent slowing in intermediate but not distal nerve segments[26]. Evidence of conduction slowing was also found to be associated with worse glycemic control in patients with type 1 diabetes[32].

The current study has several limitations. First, although only statistically significant results were considered, the patient numbers are small, especially in the CIDP+DM subgroup. In addition, misclassification and selection bias are potential errors, especially in DM patients with concomitant DSP, as there are no biomarkers to make a definitive diagnosis of CIDP. The lack of reliable biomarkers is a limitation not only for the diagnosis of CIDP, but also for assessing treatment responsiveness. Although this can be usually done reliably combining information from the clinical history, disability and functional questioners, and from clinical and electrophysiological examinations, misclassification is still a potential concern. The use of modern scales for disease severity in CIDP would have improved this study, but the scales were not available when many of these patients were evaluated. There are additional neuropathies causing proximal weakness, such as diabetic radiculoplexus neuropathies, and electrophysiological and pathologic investigations have limited capabilities for distinguishing between primary demyelinating and axonal processes[33]. Slowing of conduction velocity might also be caused by loss of ion channels in the inter-nodal region or other factors without true demyelination[26,31]. Current electrodiagnostic criteria for CIDP have also limited sensitivity, as they are research oriented, favouring specificity over sensitivity [34]. We used AAN, EFNS/PNS and study criteria with different cut off values in order to explore the association between the degree of demyelination and treatment response rates. We did not employ all 15 currently known criteria in these patients[1], or additional study criteria with additional cut off values, and so might have missed criteria with greater predictive value for treatment responsiveness. For pathological confirmation of the diagnosis, typically a sural nerve biopsy would be required, and this invasive procedure would not be well tolerated by most patients. Furthermore, pathological confirmation is not considered essential for the accurate diagnosis of CIDP, particularly since sural nerve biopsy may be misleading in CIDP[35].

In conclusion, fulfilling CIDP criteria is associated with higher treatment response rates. However, lower degrees of demyelination predict higher treatment response rates, in those patients without DM, then in those with DM. This observation might indicate that current criteria for CIDP need to be adjusted to predict treatment response rates in DM patients. An important consideration is that some patients not fulfilling the criteria examined still responded to treatment. As numbers are small, prospective studies with higher numbers of CIDP patients are required to confirm these findings, and to further explore the differences between CIDP-DM and CIDP+DM patients regarding treatment responsiveness and other factors.

## Supporting Information

S1 File Data set
